# Geometric Average Asian Option Pricing with Paying Dividend Yield under Non-Extensive Statistical Mechanics for Time-Varying Model †

**DOI:** 10.3390/e20110828

**Published:** 2018-10-28

**Authors:** Jixia Wang, Yameng Zhang

**Affiliations:** College of Mathematics and Information Science, Henan Normal University, Xinxiang 453007, Henan Province, China

**Keywords:** geometric average Asian option pricing, time-varying coefficient, Tsallis entropy distribution, Feynman–Kac formula

## Abstract

This paper is dedicated to the study of the geometric average Asian call option pricing under non-extensive statistical mechanics for a time-varying coefficient diffusion model. We employed the non-extensive Tsallis entropy distribution, which can describe the leptokurtosis and fat-tail characteristics of returns, to model the motion of the underlying asset price. Considering that economic variables change over time, we allowed the drift and diffusion terms in our model to be time-varying functions. We used the $It\hat{o}$ formula, Feynman–Kac formula, and $Pad\overset{´}{e}$ ansatz to obtain a closed-form solution of geometric average Asian option pricing with a paying dividend yield for a time-varying model. Moreover, the simulation study shows that the results obtained by our method fit the simulation data better than that of Zhao et al. From the analysis of real data, we identify the best value for *q* which can fit the real stock data, and the result shows that investors underestimate the risk using the Black–Scholes model compared to our model.

## 1. Introduction

In financial markets, the movement of asset price is the foundation of the pricing of financial assets and derivatives. Many researchers are interested in option valuation, where Black and Scholes [1] carried out milestone work. In 1973, Black and Scholes [1] constructed the Black–Scholes model to price the option, and they assumed that the empirical distributions of returns are log-normal distributions. Thereafter, many researchers continued to study option pricing based on the Black–Scholes model. For example, Wang [2] studied the Black–Scholes stock option pricing model based on dynamic investment strategy, deriving new option pricing models based on the Black–Scholes option pricing theory. Glazyrina [3] showed how the normal approximation of the binomial distribution leads to an alternative derivation of the Black–Scholes formula from a binomial option pricing model. Ulyah et al. [4] considered short-dated foreign equity options and proposed a new model based on the Black–Scholes theory for their pricing.

In the above studies, the financial markets were Brownian motion-driven models. The assumption that the price of a risky asset follows geometric Brownian motion implies that the price changes are independent. However, many papers have shown that the distribution of empirical returns does not follow log-normal distribution and has the characteristic of fat tails [5,6,7]. In subsequent papers, a number of researchers discovered that the implied volatility calculated from the Black–Scholes model shows a volatility smile. Therefore, some scholars modified the Black–Scholes model to correct for the volatility smile. For example, Hull and White [8] introduced a stochastic volatility model to correct for the smile effect. Merton [9] presented jumps to characterize the intermittent fluctuations in price changes, namely, the jump-diffusion model. Hubalek et al. [10] presented some results on geometric Asian option valuation for affine stochastic volatility models with jumps. Necula [11], Xiao [12], and Gu [13] used fractional Brownian motion with self-similarity and long-term correlation to study option pricing. However, these approaches are very complicated and cannot achieve manageable closed-form solutions.

In 1988, the Brazilian physicist Tsallis [14] proposed the non-extensive Tsallis entropy theory. Tsallis theory regards the financial market as a complex system, defines the price process of assets as an abnormal diffusion process, and obtains a simple form of a distribution function that can describe complex systems with nonlinear, long-range interactions and long-term memory effects. Recently, Tsallis theory has been widely used in the financial field. Farmer and Geanakoplos [15] pointed out that there are complex systems with asymptotic power-law behaviors in finance and economics, indicating that non-extensive statistics can be used in finance. Borland [16] used the Tsallis entropy distribution to study the European pricing issue. Ferrari [17] applied the Tsallis entropy theory to the research of value at risk, return, volatility, and asset portfolios. Based on the data from the Chinese financial market, Li [18] analyzed Tsallis entropy of the financial market. Wang et al. [19] obtained the pricing formulas of power European options based on Tsallis entropy distribution. Devi [20] obtained a good fit to a Tsallis q-Gaussian distribution for the distributions of all the returns using the method of maximum likelihood estimation. Borland [21] compared the Tsallis distribution model with the $L\overset{´}{e}vy$ model. Sosa-Correa et al. [22] found that the Tsallis distribution model performs better than the Black–Scholes model in about one-third of the option chains analyzed in the Brazilian option market.

The Asian option is one of the most active exotic options in the financial derivatives market today. In 1987, it was first introduced by the Bankers Trust in Tokyo, Japan. The difference from the standard option is that, when determining the option income on the maturity date, instead of using the current market price of the underlying asset, the Asian option uses the average of the asset price over a certain period of time during the option contract period. As Asian options are widely traded, they have received considerable attention in the financial literature. For example, Kemna and Vorst [23] proposed an expression for the geometric Asian option. Rogers and Shi [24] approached the problem of computing the price of an Asian option with the finite difference method. Benhamou and Duguet [25] presented an efficient method for pricing discrete Asian options in the presence of smile and non-proportional dividends. For geometric Asian options, Fusai and Meucci [26] studied the pricing of Asian options under the $L\overset{´}{e}vy$ process, and they provided closed-form solutions in terms of the Fourier transform. Kirkby [27] developed a method for efficiently inverting analytic characteristic functions using frame projection for geometric Asian pricing under general Levy models. Cai and Kou [28] obtained a closed-form solution for the double-Laplace transform of Asian options under the hyper-exponential jump-diffusion model. Cui et al. [29] proposed a general framework for the valuation of options in stochastic local volatility (SLV) models with a general correlation structure, and provided single Laplace transform formulae for arithmetic Asian options, as well as occupation time derivatives.

Considering that the return distribution of the underlying stock has a peak and fat tails in actual financial markets, in this study, we used the non-extensive Tsallis entropy distribution with long-term interaction and historical memory characteristics to replace the normal distribution without historical memory and modeled the motion of the underlying asset price. This model can depict the leptokurtosis and fat-tail characteristics of the distribution of returns. Furthermore, we used the $It\hat{o}$ formula, Feynman–Kac formula, and $Pad\overset{´}{e}$ ansatz to obtain a closed-form solution of geometric average Asian option pricing. Compared to Zhao’s paper [30], our method can better fit the simulation data.

The rest of this paper is organized as follows. In Section 2, we introduce the Tsallis distribution to model the price of a risky asset. In Section 3, we investigate the geometric average Asian call option and derive the pricing formula. In Section 4, we provide several simulation studies. In Section 5, we select the daily returns of a stock and perform an analysis of real data. In Section 6, we summarize our paper.

Our work differs from the existing approach of Zhao et al. [30] in the following important respects:

- In our model, we allow the coefficients of the drift term and diffusion term to be time-varying functions. In some sense, we extend the model of Zhao et al. [30].
- In this paper, we propose the geometric average Asian option pricing with paying dividend yield. So, our results are more widely applicable than that of Zhao et al. [30].
- When we derive the price formula of geometric average Asian option, the most important part is calculating the integral which is defined by Equation (13). Zhao et al. [30] got their results by using Lemma 1 in their paper. We use the Feynman–Kac formula and $Pad\overset{´}{e}$ ansatz to deal with the integral. A simulation study shows that our method can better fit the simulation data than that of Zhao et al. [30].

## 2. Model Setting

In this section, we suppose that there are stocks and bonds in the continuous financial market. W(t) is the bond price which satisfies the equation below:

(1) $$\left\{\begin{array}{c} dW(t)=r(t)W(t)dt,\\ W(0)=1, \end{array}\right.$$

where r(t) is the risk-free interest rate.

The stock price X(t) follows the time-varying coefficient stochastic process,

(2) dX(t)=μ(t)X(t)dt+σ(t)X(t)dΩ(t),

where $X\left(0\right)=X_{0}$, and μ(t) and σ(t) denote the drift term and the diffusion term, which both depend on time *t*, respectively. Also, μ(t)=r(t)−d(t), where d(t) is dividend yield.

For the probability space (Ω,F,P), we assume that Ω(·) follows the statistical feedback process

(3) $$d\Omega \left(t\right)=P{\left(\Omega \left(t\right)\right)}^{\frac{1-q}{2}}dB\left(t\right),$$

where B(t) is the standard Brownian motion. The probability distribution of Ω(t) derives from the nonlinear Fokker–Planck equation,

$$\frac{\partial }{\partial t}P\left(\Omega \left(t\right),t\right)=\frac{1}{2}\frac{\partial^{2}}{\partial \Omega {\left(t\right)}^{2}}P^{2-q}\left(\Omega \left(t\right),t\right).$$

The probability density function P(Ω(t),t) is called the Tsallis distribution (see [30]), which is given by

$$P\left(\Omega \left(t\right),t\right)=\frac{1}{Z(t)}{\left(1-\beta \left(t\right)\left(1-q\right)\Omega {\left(t\right)}^{2}\right)}^{\frac{1}{1-q}},$$

with

$$\begin{array}{c} \beta \left(t\right)=k^{\frac{1-q}{3-q}}{\left((2-q)(3-q)t\right)}^{\frac{-2}{3-q}},\\ Z\left(t\right)=\int_{-\infty }^{+\infty }{\left(1-\left(1-q\right)\beta \left(t\right)\Omega {\left(t\right)}^{2}\right)}^{\frac{1}{1-q}}\;d\Omega \left(t\right)={\left((2-q)(3-q)kt\right)}^{\frac{1}{3-q}},\\ k=\frac{\pi }{q-1}\frac{\Gamma^{2}\left(\frac{1}{q-1}-\frac{1}{2}\right)}{\Gamma^{2}\left(\frac{1}{q-1}\right)}, \end{array}$$

where Γ(·) is the gamma function.

It is easy to calculate that its mean is zero, and its variance is

(4) $$Var\left(\Omega \left(t\right)\right)=E\left[\Omega {\left(t\right)}^{2}\right]=\frac{1}{\left(5-3q)\beta (t\right)}.$$

According to Equation (4), the variance diverges for $q>\frac{5}{3}$. Thus, we only consider $\frac{5}{3}>q>1$, for which the variance is limited.

Then, we define an equivalent martingale measure Q. Let

(5) $$\begin{array}{c} \theta \left(t\right)=\frac{\mu (t)-r(t)}{\sigma \left(t\right)P^{\frac{1-q}{2}}},\\ \tilde{B}\left(t\right)=B\left(t\right)+\int_{0}^{t}\theta \left(s\right)\;ds, \end{array}$$

where $P^{\frac{1-q}{2}}=P{\left(\Omega \left(t\right)\right)}^{\frac{1-q}{2}}$ and $E[\exp\left(\frac{1}{2}\int_{0}^{t}\theta^{2}\left(s\right)\;ds\right)]<\infty$ (see [31]). We construct the Radon–Nikodým derivative between the equivalent martingale measures Q and P as follows

$$\frac{dQ}{dP}=\exp\left(\int_{0}^{t}\theta \left(s\right)\;dB\left(s\right)-\frac{1}{2}\int_{0}^{t}\theta^{2}\left(s\right)\;ds\right).$$

Under the probability measure Q, $\tilde{B}\left(t\right)$ is the standard Brownian motion, which can be derived from Girsanov theorem.

In order to calculate the stock price, we need to prove that the discounted stock price process

(6) $$X^{*}\left(t\right)=e^{-\int_{0}^{t}r\left(s\right)\;ds}X\left(t\right)$$

is a martingale under the probability measure Q.

In fact, applying Equation (5) to Equation (2), we obtain

(7) $$\begin{array}{cc} dX(t) & =\mu \left(t\right)X\left(t\right)dt+\sigma \left(t\right)X\left(t\right)P^{\frac{1-q}{2}}dB\left(t\right)\\ & =\mu \left(t\right)X\left(t\right)dt+\sigma \left(t\right)X\left(t\right)P^{\frac{1-q}{2}}\left(d\tilde{B}\left(t\right)-\theta \left(t\right)dt\right)\\ & =\sigma \left(t\right)X\left(t\right)P^{\frac{1-q}{2}}d\tilde{B}\left(t\right)+r\left(t\right)X\left(t\right)dt. \end{array}$$

Using the $It\hat{o}$ formula on Equation (6), we get

$$dX^{*}\left(t\right)=e^{-\int_{0}^{t}r\left(s\right)\;ds}dX\left(t\right)+\left(-r\left(t\right)e^{-\int_{0}^{t}r\left(s\right)\;ds}\right)X\left(t\right)dt.$$

Substituting Equation (7),

$$\begin{array}{cc} dX^{*}\left(t\right) & =e^{-\int_{0}^{t}r\left(s\right)\;ds}\left(\sigma \left(t\right)X\left(t\right)P^{\frac{1-q}{2}}d\tilde{B}\left(t\right)+r\left(t\right)X\left(t\right)dt\right)\\ & \;\;\;+\left(-r\left(t\right)e^{-\int_{0}^{t}r\left(s\right)\;ds}\right)X\left(t\right)dt\\ & =e^{-\int_{0}^{t}r\left(s\right)\;ds}\sigma \left(t\right)X\left(t\right)P^{\frac{1-q}{2}}d\tilde{B}\left(t\right)\\ & =X^{*}\left(t\right)\sigma \left(t\right)X\left(t\right)P^{\frac{1-q}{2}}d\tilde{B}\left(t\right). \end{array}$$

Based on the above discussion, $X^{*}\left(t\right)$ is a martingale under the probability measure Q.

Theorem 1 below gives the solution of Equation (2).

**Theorem** **1.** *Under the measure Q, we obtain the approximate value of the solution of Equation (2) as below*

(8) $$\begin{array}{cc} X(t)= & X\left(0\right)\exp[\int_{0}^{t}r\left(s\right)\;ds+\sigma \left(t\right)\Omega \left(t\right)-\int_{0}^{t}\frac{1}{2}\sigma {\left(s\right)}^{2}Z{\left(s\right)}^{q-1}\;ds\\ & +\frac{1}{2}\left(1-q\right)\left(g_{0}\left(t\right)+g_{2}\left(t\right)\Omega {\left(t\right)}^{2}\right)]. \end{array}$$

*where*

(9) $$\begin{array}{cc} g_{0}\left(t\right)= & \int_{0}^{t}Z{\left(s\right)}^{q-1}g_{2}\left(s\right)\;ds\\ g_{2}\left(t\right)= & \frac{t^{\frac{5q-9}{\left(2-q)(3-q\right)}}}{\left(2-q)(3-q\right)}\int_{0}^{t}\sigma {\left(s\right)}^{2}s^{-1-\frac{5q-9}{\left(2-q)(3-q\right)}}\;ds. \end{array}$$

**Proof.**  According to Equation (2), we can get

(10) $$dX\left(t\right)=\left(\mu \left(t\right)dt+\sigma \left(t\right)P^{\frac{1-q}{2}}dB\left(t\right)\right)X\left(t\right).$$

Applying the It $\hat{o}$ formula to lnX(t), we obtain

(11) $$d\ln X\left(t\right)=\frac{1}{X(t)}dX\left(t\right)-\frac{1}{2}\sigma^{2}\left(t\right)P^{1-q}dt.$$

Substituting Equation (7), we have

(12) $$d\ln X\left(t\right)=\sigma \left(t\right)P^{\frac{1-q}{2}}d\tilde{B}\left(t\right)+\left(r\left(t\right)-\frac{1}{2}\sigma^{2}\left(t\right)P^{1-q}\right)dt.$$

Integrating both sides of Equation (12) simultaneously in [0,t],

$$\ln X\left(t\right)-\ln X\left(0\right)=\int_{0}^{t}\sigma \left(s\right)P^{\frac{1-q}{2}}\;d\tilde{B}\left(s\right)+\int_{0}^{t}\left(r\left(s\right)-\frac{1}{2}\sigma^{2}\left(s\right)P^{1-q}\right)\;ds.$$

Consequently, we infer that

$$\begin{array}{ccc} X(t) & = & X\left(0\right)\exp\left[\int_{0}^{t}\sigma \left(s\right)P^{\frac{1-q}{2}}\;d\tilde{B}\left(s\right)+\int_{0}^{t}\left(r\left(s\right)-\frac{1}{2}\sigma^{2}\left(s\right)P^{1-q}\right)\;ds\right]\\ & = & X\left(0\right)\exp[\sigma \left(t\right)\Omega \left(t\right)-\int_{0}^{t}\frac{1}{2}\sigma {\left(s\right)}^{2}Z{\left(s\right)}^{q-1}\left(1-\beta \left(s\right)\left(1-q\right)\Omega {\left(s\right)}^{2}\right)\;ds\\ & & +\int_{0}^{t}r\left(s\right)\;ds]\\ & = & X\left(0\right)\exp[\int_{0}^{t}r\left(s\right)\;ds+\sigma \left(t\right)\Omega \left(t\right)-\int_{0}^{t}\frac{1}{2}\sigma {\left(s\right)}^{2}Z{\left(s\right)}^{q-1}\;ds\\ & & +\frac{1}{2}\left(1-q\right)\int_{0}^{t}\sigma {\left(s\right)}^{2}Z{\left(s\right)}^{q-1}\beta \left(s\right)\Omega {\left(s\right)}^{2}\;ds]\\ & = & X\left(0\right)\exp[\int_{0}^{t}r\left(s\right)\;ds+\sigma \left(t\right)\Omega \left(t\right)-\int_{0}^{t}\frac{1}{2}\sigma {\left(s\right)}^{2}Z{\left(s\right)}^{q-1}\;ds\\ & & +\frac{1}{2}\left(1-q\right)I\left(t\right)]. \end{array}$$

where

(13) $$I\left(t\right)=\int_{0}^{t}\sigma {\left(s\right)}^{2}Z{\left(s\right)}^{q-1}\beta \left(s\right)\Omega {\left(s\right)}^{2}\;ds.$$

It is hard to calculate the integral I(t), which contains the term of the type $\int_{0}^{t}F\left(\Omega \left(s\right)\right)\;ds$. Thus, we use the Feynman–Kac formula and the $Pad\overset{´}{e}$ ansatz, which are valid according to [31], to evaluate I(t).

First, we define $U\left(\Omega \left(T\right),T\right)=\sum_{i|\Omega (T)}\omega_{i}\exp\left\{\varepsilon \int_{0}^{T}F\left(\Omega \left(t\right)\right)\;dt\right\}$, where $\omega_{i}$ is the weight of a path *i* ending at Ω(T) at time *T*, and ε is a small parameter. Then, we expand the polynomial to the second order in Ω,

(14) $$U\left(\Omega \left(T\right),T\right)=\sum_{i|\Omega (T)}\omega_{i}\exp\left[\varepsilon \int_{0}^{T}\left(\sum_{j=1}^{2}f_{j}\left(t\right)\Omega {\left(t\right)}^{j}\right)\;dt\right].$$

We establish a generalized Feynman–Kac equation as follows:

(15) $$\frac{\partial U}{\partial T}=\frac{1}{2}\frac{\partial^{2}U_{0}^{1-q}U}{\partial \Omega^{2}}+\varepsilon \sum_{j}f_{j}\left(T\right)\Omega {\left(T\right)}^{j}.$$

When we insert the ansatz $U=U_{0}\left[1+\varepsilon \left(g_{0}\left(T\right)+g_{1}\left(T\right)\Omega \left(T\right)+g_{2}\left(T\right)\Omega {\left(T\right)}^{2}\right)\right]$ into Equation (15), the coefficients $g_{i}$ must satisfy

(16) $$\begin{array}{cc} \frac{dg_{0}}{dt}= & Z{\left(t\right)}^{q-1}g_{2}\left(t\right)\\ \frac{dg_{1}}{dt}= & 2\left(q-2\right)Z{\left(t\right)}^{q-1}\beta \left(t\right)g_{1}\left(t\right)+h_{1}\left(t\right)\\ \frac{dg_{2}}{dt}= & \left(5q-9\right)Z{\left(t\right)}^{q-1}\beta \left(t\right)g_{2}\left(t\right)+h_{2}\left(t\right). \end{array}$$

For I(t), we have $h_{1}=0,h_{2}=\sigma {\left(t\right)}^{2}Z{\left(t\right)}^{q-1}\beta \left(t\right)$. The coefficients $g_{i}$ are calculated from Equation (16):

(17) $$\begin{array}{cc} g_{0}\left(t\right)= & \int_{0}^{t}Z{\left(s\right)}^{q-1}g_{2}\left(s\right)\;ds\\ g_{2}\left(t\right)= & \frac{t^{\frac{5q-9}{\left(2-q)(3-q\right)}}}{\left(2-q)(3-q\right)}\int_{0}^{t}\sigma {\left(s\right)}^{2}s^{-1-\frac{5q-9}{\left(2-q)(3-q\right)}}\;ds. \end{array}$$

Next, we can get $I\left(t\right)=g_{0}\left(t\right)+g_{2}\left(t\right)\Omega {\left(t\right)}^{2}$. Finally, we substitute the terms of X(t) for I(t),

(18) $$\begin{array}{cc} X(t)= & X\left(0\right)\exp[\int_{0}^{t}r\left(s\right)\;ds+\sigma \left(t\right)\Omega \left(t\right)-\int_{0}^{t}\frac{1}{2}\sigma {\left(s\right)}^{2}Z{\left(s\right)}^{q-1}\;ds\\ & +\frac{1}{2}\left(1-q\right)\left(g_{0}\left(t\right)+g_{2}\left(t\right)\Omega {\left(t\right)}^{2}\right)]. \end{array}$$

This completes the proof of Theorem 1. □

## 3. Geometric Average Asian Option Pricing Formula

In this section, we discuss the geometric average Asian call option pricing. Suppose that the maturity time is *T*, the strike price is *K*, and $J_{T}=\exp\left(\frac{1}{T}\int_{0}^{T}\ln X\left(t\right)\;dt\right)$, which is the average price. It is well known that a geometric average Asian call option price can be written as below (see [32]):

(19) $$C=e^{-\int_{0}^{T}r\left(t\right)\;dt}E\left[{\left(J_{T}-K\right)}^{+}\right].$$

**Theorem** **2.** *Given a geometric average Asian call option, it has the following payoff at the maturity time T:*

(20) $${\left(J_{T}-K\right)}^{+}$$

*where K is the strike price, and $J_{T}=\exp\left(\frac{1}{T}\int_{0}^{T}\ln X\left(t\right)\;dt\right)$. Then, the approximation of the option price in the risk-neutral world is given as below:*

(21) $$\begin{array}{cc} C= & e^{-\int_{0}^{T}\left(r\left(t\right)-d\left(t\right)\right)\;dt}\left\{X\left(0\right)\int_{\omega_{1}}^{\omega_{2}}e^{\frac{c}{T}+\frac{g_{3}\left(t\right)}{T}\Omega \left(t\right)+\frac{1-q}{2T}g_{5}\left(t\right)\Omega {\left(t\right)}^{2}}P\left(\Omega ,T\right)\;d\Omega \right\}\\ & -e^{-\int_{0}^{T}r\left(t\right)\;dt}K\int_{\omega_{1}}^{\omega_{2}}P\left(\Omega ,T\right)\;d\Omega , \end{array}$$

*where*

(22) $$\begin{array}{cc} c= & \int_{0}^{T}\int_{0}^{t}r\left(s\right)\;ds\;dt-\int_{0}^{T}\int_{0}^{t}\frac{1}{2}\sigma {\left(s\right)}^{2}Z{\left(s\right)}^{q-1}\;ds\;dt+\frac{1}{2}\left(1-q\right)g_{4}\left(t\right)+\int_{0}^{T}\frac{1}{2}\left(1-q\right)g_{0}\left(t\right)\;dt\\ g_{0}= & \int_{0}^{t}Z{\left(s\right)}^{q-1}g_{2}\left(s\right)\;ds\\ g_{2}= & \frac{t^{\frac{5q-9}{\left(2-q)(3-q\right)}}}{\left(2-q)(3-q\right)}\int_{0}^{t}\sigma {\left(s\right)}^{2}s^{-1-\frac{5q-9}{\left(2-q)(3-q\right)}}\;ds\\ g_{3}= & t^{-\frac{2}{3-q}}\int_{0}^{t}\sigma \left(s\right)s^{\frac{2}{3-q}}\;ds\\ g_{4}= & \frac{1}{\left(2-q)(3-q\right)}\int_{0}^{t}Z{\left(s\right)}^{q-1}g_{2}\left(s\right)\;ds\\ g_{5}= & \frac{t^{\frac{5q-9}{\left(2-q)(3-q\right)}}}{\left(2-q)(3-q\right)}\int_{0}^{t}\int_{0}^{u}\sigma {\left(s\right)}^{2}s^{-1-\frac{5q-9}{\left(2-q)(3-q\right)}}\;ds\;du\\ \omega_{1}= & \frac{-g_{3}\left(t\right)-\sqrt{\Delta }}{\left(1-q\right)g_{5}\left(t\right)}\\ \omega_{2}= & \frac{-g_{3}\left(t\right)+\sqrt{\Delta }}{\left(1-q\right)g_{5}\left(t\right)}\\ \Delta = & g_{3}{\left(t\right)}^{2}-4\left(\frac{1}{2}\left(1-q\right)g_{5}\left(t\right)\right)\left(c+T\ln\frac{X(0)}{K}\right). \end{array}$$

**Proof.**  According to Equation (19), we can obtain

(23) $$\begin{array}{cc} C & =e^{-\int_{0}^{T}r\left(t\right)\;dt}E{\left[J_{T}\right]}_{\left\{J_{T}>K\right\}}-e^{-\int_{0}^{T}r\left(t\right)\;dt}E{\left[K\right]}_{\left\{J_{T}>K\right\}}\\ & =M-N, \end{array}$$

where

(24) $$\begin{array}{c} M:=e^{-\int_{0}^{T}r\left(t\right)\;dt}E{\left[J_{T}\right]}_{\left\{J_{T}>K\right\}}\\ N:=e^{-\int_{0}^{T}r\left(t\right)\;dt}E{\left[K\right]}_{\left\{J_{T}>K\right\}}. \end{array}$$

Then, we should calculate the domain of inequality $\left\{J_{T}>K\right\}$, which is equal to $\exp\left(\frac{1}{T}\int_{0}^{T}\ln X\left(t\right)\;dt\right)>K$. It follows that

$$\int_{0}^{T}\ln X\left(t\right)\;dt>T\ln K.$$

Using Theorem 1, we plug X(t) back into the above inequality,

$$\begin{array}{c} T\ln X\left(0\right)+\int_{0}^{T}\int_{0}^{t}r\left(s\right)\;ds\;dt+\int_{0}^{T}\sigma \left(t\right)\Omega \left(t\right)\;dt+\int_{0}^{T}\frac{1}{2}\left(1-q\right)g_{0}\left(t\right)\;dt\\ -\int_{0}^{T}\int_{0}^{t}\frac{1}{2}\sigma {\left(s\right)}^{2}Z{\left(s\right)}^{q-1}\;ds\;dt+\int_{0}^{T}\frac{1}{2}\left(1-q\right)g_{2}\left(t\right)\Omega {\left(t\right)}^{2}\;dt>T\ln K. \end{array}$$

After the computation, we get

(25) $$\begin{array}{c} T\ln X\left(0\right)+\int_{0}^{T}\int_{0}^{t}r\left(s\right)\;ds\;dt-\int_{0}^{T}\int_{0}^{t}\frac{1}{2}\sigma {\left(s\right)}^{2}Z{\left(s\right)}^{q-1}\;ds\;dt-T\ln K\\ +\int_{0}^{T}\frac{1}{2}\left(1-q\right)g_{0}\left(t\right)\;dt+\int_{0}^{T}\sigma \left(t\right)\Omega \left(t\right)\;dt+\frac{1}{2}\left(1-q\right)\int_{0}^{T}g_{2}\left(t\right)\Omega {\left(t\right)}^{2}\;dt>0. \end{array}$$

For the convenience of calculation, we denote the following:

(26) $$\begin{array}{cc} A= & \int_{0}^{T}\sigma \left(t\right)\Omega \left(t\right)\;dt\\ B= & \int_{0}^{T}g_{2}\left(t\right)\Omega {\left(t\right)}^{2}\;dt. \end{array}$$

We use the Feynman–Kac approach and the $Pad\overset{´}{e}$ ansatz to evaluate *A* and *B*, for *A* and *B* both have the terms of $\int_{0}^{t}F\left(\Omega \left(s\right)\right)\;ds$.

For *A*, the coefficients $g_{i}$ are calculated from Equation (16) with $h_{1}=\sigma \left(s\right)$:

(27) $$g_{1}\left(t\right)=t^{-\frac{2}{3-q}}\int_{0}^{t}\sigma \left(s\right)s^{\frac{2}{3-q}}\;ds.$$

To avoid confusion, we let $g_{3}=g_{1}$ and get

(28) $$A=g_{3}\left(t\right)\Omega \left(t\right).$$

For *B*, we have $h_{0}=0,h_{2}=\frac{t^{\frac{5q-9}{\left(2-q)(3-q\right)}}}{\left(2-q)(3-q\right)}\int_{0}^{t}\sigma {\left(s\right)}^{2}s^{-1-\frac{5q-9}{\left(2-q)(3-q\right)}}\;ds$. Then, we can calculate the coefficients $g_{i}$ from Equation (16), as below:

(29) $$\begin{array}{cc} g_{0}\left(t\right)= & \frac{1}{\left(2-q)(3-q\right)}\int_{0}^{t}Z{\left(s\right)}^{q-1}g_{2}\left(s\right)\;ds\\ g_{2}\left(t\right)= & \frac{t^{\frac{5q-9}{\left(2-q)(3-q\right)}}}{\left(2-q)(3-q\right)}\int_{0}^{t}\int_{0}^{u}\sigma {\left(s\right)}^{2}s^{-1-\frac{5q-9}{\left(2-q)(3-q\right)}}\;ds\;du. \end{array}$$

In order to make the expression clearer, we allow $g_{4}=g_{0},g_{5}=g_{2}$ and get

(30) $$B=g_{4}\left(t\right)+g_{5}\left(t\right)\Omega {\left(t\right)}^{2}.$$

According to the above approximations, we replace the terms of Equation (25) with *A* and *B*,

(31) $$\frac{1}{2}\left(1-q\right)g_{5}\left(t\right)\Omega {\left(t\right)}^{2}+g_{3}\left(t\right)\Omega \left(t\right)+c+T\ln\frac{X(0)}{K}>0,$$

where

(32) $$\begin{array}{cc} c= & \int_{0}^{T}\int_{0}^{t}r\left(s\right)\;ds\;dt-\int_{0}^{T}\int_{0}^{t}\frac{1}{2}\sigma {\left(s\right)}^{2}Z{\left(s\right)}^{q-1}\;ds\;dt+\frac{1}{2}\left(1-q\right)g_{4}\left(t\right)\\ & +\int_{0}^{T}\frac{1}{2}\left(1-q\right)g_{0}\left(t\right)\;dt. \end{array}$$

Because $\frac{5}{3}>q>1$, it is clear that $\frac{1}{2}\left(1-q\right)g_{5}\left(t\right)<0$.

Let $K<\exp\left(\ln X(0)-\frac{1}{T}\left(\frac{g_{3}{\left(t\right)}^{2}}{2\left(1-q\right)g_{5}\left(t\right)}-c\right)\right)$; then, we can obtain

$$\Delta =g_{3}{\left(t\right)}^{2}-4\left(\frac{1}{2}\left(1-q\right)g_{5}\left(t\right)\right)\left(c+T\ln\frac{X(0)}{K}\right)>0.$$

Therefore, the quadratic inequality’s corresponding quadratic equation has two roots,

$$\omega_{1}=\frac{-g_{3}\left(t\right)-\sqrt{\Delta }}{\left(1-q\right)g_{5}\left(t\right)},$$

and

$$\omega_{2}=\frac{-g_{3}\left(t\right)+\sqrt{\Delta }}{\left(1-q\right)g_{5}\left(t\right)}.$$

Hence, we get the solution set of $\left\{J_{T}>K\right\}$ as $\omega \in (\omega_{1},\omega_{2})$. Then, we can calculate *M* and *N* in the risk-neutral world as follows

$$\begin{array}{cc} M & =e^{-\int_{0}^{T}r\left(t\right)\;dt}E{\left[J_{T}\right]}_{\left\{J_{T}>K\right\}}\int_{\omega_{1}}^{\omega_{2}}\;d\Omega \\ & =X\left(0\right)e^{-\int_{0}^{T}\left(r\left(t\right)-d\left(t\right)\right)\;dt}\int_{\omega_{1}}^{\omega_{2}}e^{\frac{c}{T}+\frac{g_{3}\left(t\right)}{T}\Omega \left(t\right)+\frac{1-q}{2T}g_{5}\left(t\right)\Omega {\left(t\right)}^{2}}P\left(\Omega ,T\right)\;d\Omega . \end{array}$$

Similar to *M*, we obtain

$$\begin{array}{cc} N & =e^{-\int_{0}^{T}r\left(t\right)\;dt}E{\left[K\right]}_{\left\{J_{T}>K\right\}}\int_{\omega_{1}}^{\omega_{2}}\;d\Omega \\ & =e^{-\int_{0}^{T}r\left(t\right)\;dt}K\int_{\omega_{1}}^{\omega_{2}}P\left(\Omega ,T\right)\;d\Omega . \end{array}$$

Hence, we get the option price as follows:

$$\begin{array}{ccc} C & = & M-N\\ & = & e^{-\int_{0}^{T}\left(r\left(t\right)-d\left(t\right)\right)\;dt}\left\{X\left(0\right)\int_{\omega_{1}}^{\omega_{2}}e^{\frac{c}{T}+\frac{g_{3}\left(t\right)}{T}\Omega \left(t\right)+\frac{1-q}{2T}g_{5}\left(t\right)\Omega {\left(t\right)}^{2}}P\left(\Omega ,T\right)\;d\Omega \right\}\\ & & -e^{-\int_{0}^{T}r\left(t\right)\;dt}K\int_{\omega_{1}}^{\omega_{2}}P\left(\Omega ,T\right)\;d\Omega . \end{array}$$

This completes the proof of Theorem 2. □

## 4. Simulation Study

In this section, the results of a simulation study are presented to show the difference between our method and the method of Zhao [30]. In order to compare with Zhao, we assume that r=0.5,σ=0.25. Without loss of generality, we can take μ=0.

When we calculate the geometric average Asian option price, it is hard to solve the terms of $\int_{0}^{T}\beta \left(t\right)Z{\left(t\right)}^{q-1}\Omega {\left(t\right)}^{2}\;dt$ since the Ω(t) in the integral function is a stochastic process. We used the Feynman–Kac formula and $Pad\overset{´}{e}$ ansatz to solve them, while Zhao et al. [30] introduced the transformation $\Omega \left(t\right)=\sqrt{\frac{\beta (T)}{\beta (t)}}\Omega \left(T\right)$. In order to compare the two methods, we paint a picture of $\int_{0}^{T}\beta \left(t\right)Z{\left(t\right)}^{\left(q-1\right)}\Omega {\left(t\right)}^{2}\;dt$ versus Ω(T) in the following three ways:

(1) Monte-Carlo simulation;
(2) $\Omega \left(t\right)=\sqrt{\frac{\beta (T)}{\beta (t)}}\Omega \left(T\right)$ in Zhao et al. [30];
(3) Feynman–Kac formula and $Pad\overset{´}{e}$ ansatz method in our paper.

**Figure 1:**
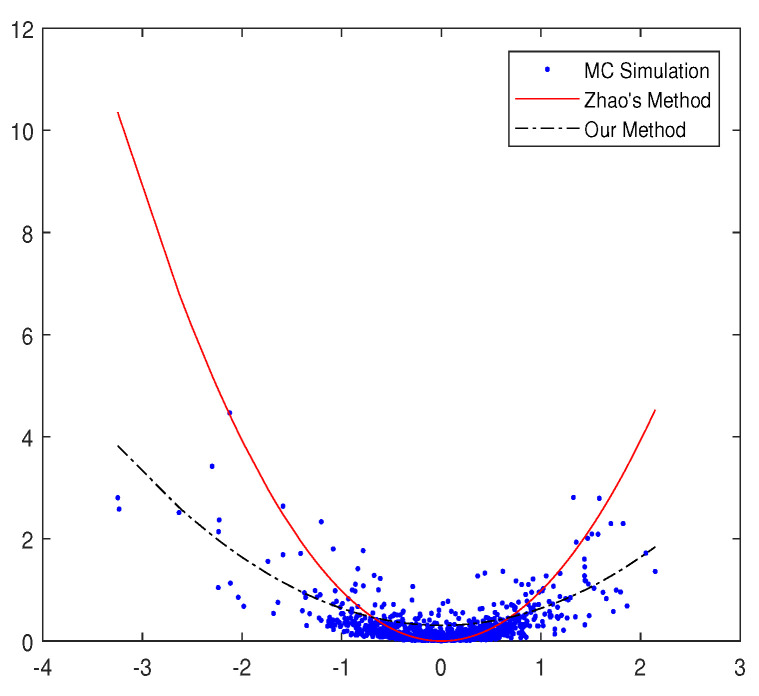
The *x*-axis is Ω(T), and the *y*-axis is $\int_{0}^{T}\beta \left(t\right)Z{\left(t\right)}^{\left(q-1\right)}\Omega {\left(t\right)}^{2}\;dt$. The points were obtained by Monte-Carlo simulation, the red line is calculated in Zhao’s paper, and the black line is obtained by the Feynman–Kac formula and $Pad\overset{´}{e}$ ansatz in our paper.

Assuming T=0.5,q=1.5, then we get the following figure. It is also discussed in the appendix of [31]. In Figure 1, it is obvious that the black line fits the discrete points better than the red one. In other words, the data obtained by our method are closer to the simulation data of the real market. The black line is extremely close to those points obtained by Monte-Carlo simulation. From Figure 1, the data calculated by the Feynman–Kac formula and $Pad\overset{´}{e}$ ansatz are more responsive to the real market data than the method obtained by Zhao et al. [30]. 

Next, we describe some simulation studies to show the difference between our model and the Black–Scholes model. According to Michael and Johnson’s work [33], the data generated by the Tsallis distribution can describe the real market data accurately, so we used the Tsallis distribution to generate the simulation data. Some steps of the numerical simulation are given as follows:

Step 1: We assume the value of q,K,r,σ,T, etc.
Step 2: We generate 1000 random numbers of the Tsallis distribution.
Step 3: According to step 2, we can calculate the price of a risky asset by Equation (8).
Step 4: We derive the geometric average Asian call option price by Equation (21) and generate the resulting figures.

Before examining the numerical simulation, we first give the pricing formula of the Black–Scholes model, which is

(33) $$P=X\left(0\right)e^{-\frac{1}{2}\int_{0}^{T}\left(r\left(t\right)+\frac{1}{6}\sigma {\left(t\right)}^{2}\right)\;dt}N\left(d_{1}\right)-Ke^{-\int_{0}^{T}r\left(t\right)\;dt}N\left(d_{2}\right)$$

where

(34) $$\begin{array}{c} d_{1}=\frac{\ln\frac{X(0)}{K}+\frac{1}{2}\int_{0}^{T}\left(r\left(t\right)+\frac{1}{6}\sigma {\left(t\right)}^{2}\right)\;dt}{\sqrt{\frac{3}{T}\int_{0}^{T}\sigma {\left(t\right)}^{2}\;dt}},\;\;d_{2}=d_{1}-\sqrt{\frac{3}{T}\int_{0}^{T}\sigma {\left(t\right)}^{2}\;dt} \end{array}$$

**Figure 2:**
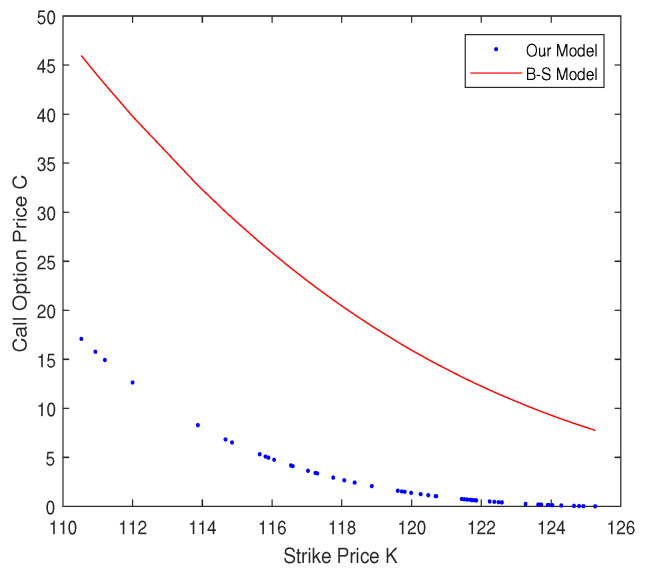
The *x*-axis is the strike price *K*, and the *y*-axis is the geometric average Asian call option price *C*.

Let X(0)=100. Without loss of generality, we used d(t)=0. Figure 2 depicts the difference between our model and the Black–Scholes model.

In Figure 2, the call option prices are becoming lower as the strike price *K* becomes larger. Moreover, the call option price calculated by our model is lower than that from the Black–Scholes model. This suggests that investors underestimate the risk using the Black–Scholes model. According to Figure 2, we can see that our model is a better fit than the Black–Scholes model.

## 5. Analysis of Real Data

In this section, we use actual cases to test the model. We selected the daily closing prices of a stock with a code of “601318” (hereinafter called “601318”) in the Chinese stock market as real data. The stock is issued by the Ping An Insurance (Group) Company of China. The time period is from 1 March 2007 to 1 March 2018 and the sample size is 2612.

In Table 1, “J-B” is the value of the Jarque–Bera test, which is a test for normality comparing sample skewness and kurtosis against the theoretical values for a normal distribution. “P” is the *p*-value associated with this test. From the basic statistical values in Table 1, it can be seen that the daily returns of “601318” have obvious characteristics of leptokurtosis and of fat tail. The kurtosis of the empirical data is 200.9483, and it is well known that the kurtosis of a normal distribution is 3. Comparing these values of kurtosis, 200.9483 is much larger than 3. The estimated kurtosis of 200.9483 demonstrates that the empirical tails are much heavier than those of a normal distribution, which has a kurtosis of 3. Therefore, it is not appropriate to describe real data with a normal distribution. The *p*-value corresponding the Jarque–Berra test of normality leads us to reject the null hypothesis of normality for this sample.

**Figure 3:**
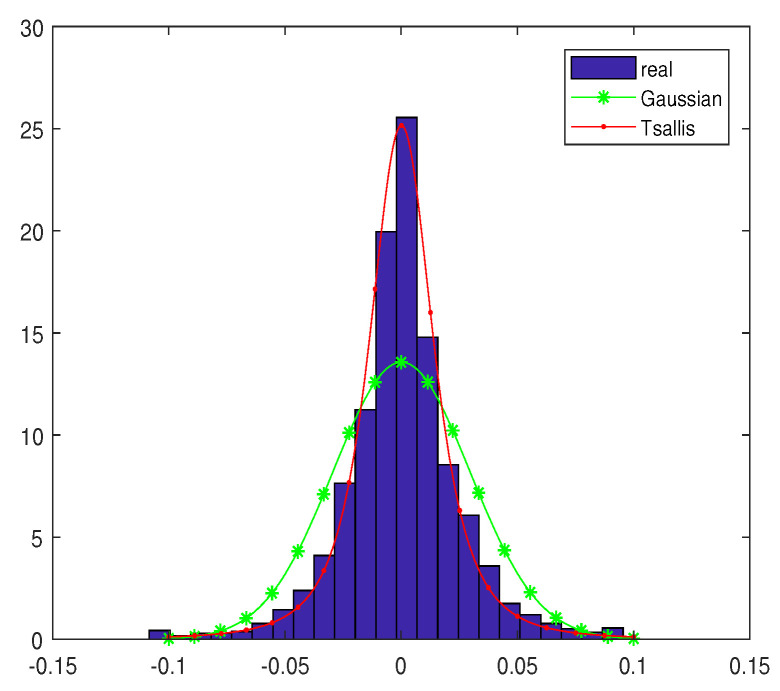
Comparison of the fitting of the empirical distribution of the daily returns for the empirical distribution, normal distribution, and q-Gaussian distribution.

In Figure 3, we compare the fitting of the empirical distribution of the daily returns for the empirical distribution, normal distribution, and q-Gaussian distribution with q=1.55. We can see that the Gaussian distribution cannot describe the peak of the empirical data. According to the calculation results and several experiments, we discover that a Tsallis distribution with the parameter q=1.55 can fit the empirical density distribution of daily returns more accurately than the normal distribution.

**Figure 4:**
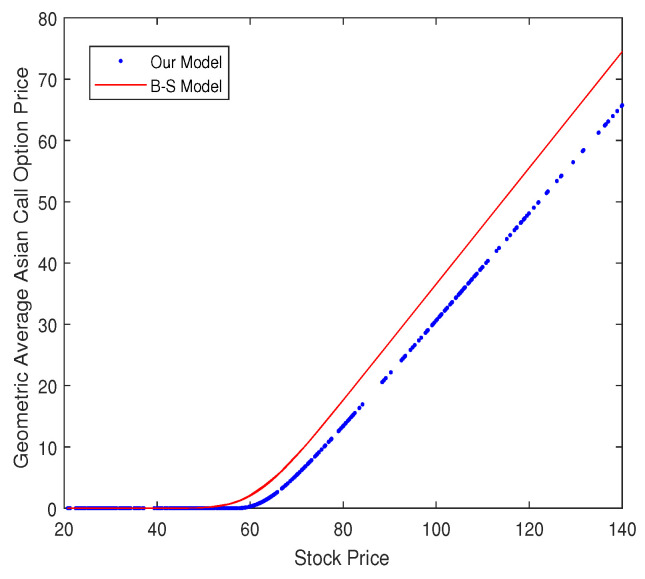
Comparison of the geometric average Asian call option price for our model and the Black–Scholes model.

Figure 4 shows the difference in the geometric average Asian call option price between our model and the Black–Scholes model. The data are the stock prices of “601318”. In the graph, we can observe that the price calculated by the Black–Scholes model is higher than that of our model. Theoretically speaking, it is because the tail of the distribution of the stock price is heavy. This suggests that the investors overestimate the option price using the Black–Scholes model.

## 6. Summary

This paper mainly examines the geometric average Asian call option pricing under the time-varying coefficient diffusion model. The underlying asset price is modeled by using the non-extensive Tsallis entropy distribution. Considering that economic variables change from time to time, we allow both drift and diffusion terms in our model to be time-varying functions. We obtain the closed-form solution of geometric average Asian option pricing with paying dividend yield for the time-varying model by using the $It\hat{o}$ formula, Feynman–Kac formula, and $Pad\overset{´}{e}$ ansatz. Moreover, the simulation studies show that the results obtained by our method fit the real data better than the method of Zhao et al. [30]. Also, investors estimate the risk more reasonably using our model than with the Black–Scholes model. Based on the results obtained through the analysis of real data and reported in our paper, we determine that q=1.55 is the optimal *q* value to fit the real stock data.

## Figures and Tables

**Table 1:** The basic statistics of daily returns of “601318”.

Sample Size	Mean	Std	Kurtosis	J–B	P
2612	1.4838 × 10${}^{-4}$	8.7055 × 10${}^{-4}$	200.9483	4.2867 × 10${}^{6}$	0
